# Volume transmission signalling via astrocytes

**DOI:** 10.1098/rstb.2013.0604

**Published:** 2014-10-19

**Authors:** Hajime Hirase, Youichi Iwai, Norio Takata, Yoshiaki Shinohara, Tsuneko Mishima

**Affiliations:** 1 Laboratory for Neuron-Glia Circuitry, RIKEN Brain Science Institute, Wako, Saitama, Japan; 2 Saitama University Brain Science Institute, Saitama, Saitama, Japan; 3 Department of Neuropsychiatry, School of Medicine, Keio University, Shinjuku, Tokyo, Japan

**Keywords:** acetylcholine, Gq signalling, IP_3_ receptors, d-serine, gamma oscillations

## Abstract

The influence of astrocytes on synaptic function has been increasingly studied, owing to the discovery of both gliotransmission and morphological ensheathment of synapses. While astrocytes exhibit at best modest membrane potential fluctuations, activation of G-protein coupled receptors (GPCRs) leads to a prominent elevation of intracellular calcium which has been reported to correlate with gliotransmission. In this review, the possible role of astrocytic GPCR activation is discussed as a trigger to promote synaptic plasticity, by affecting synaptic receptors through gliotransmitters. Moreover, we suggest that volume transmission of neuromodulators could be a biological mechanism to activate astrocytic GPCRs and thereby to switch synaptic networks to the plastic mode during states of attention in cerebral cortical structures.

## 1. Introduction

With the advent of molecular genetics and cellular imaging techniques, our understanding of brain function has advanced substantially in the recent decade. Glial cell research has indisputably benefited from these techniques, as glial cells are generally electrically passive, and their dynamism resides most probably in biochemical and morphological changes. Among the glial cells, astrocytes occupy a significant proportion of the brain volume in mammals and are arguably the most numerous in primate cortical grey matter. The morphology of astrocytes is best described as an interface between vascular and neuronal networks. A typical protoplasmic astrocyte has a bushy organization of microprocesses that surround synapses and a few large processes that impinge on neighbouring vasculature (giant end-feet). For white matter fibrous astrocytes, the microprocesses extend around the nodal regions of myelinated axons. Such strategic positioning of astrocytes is indeed well matched with the classically supposed functions of astrocytes, including the clearance of synaptically released neurotransmitters, regulation of ionic concentrations and mediation of energy metabolism substrates. Since gliotransmission—the ability of astrocytes to secrete biochemical molecules to influence surrounding neurons—was discovered about two decades ago [1–3], astrocytes have been hypothesized to play active roles in neuronal network operations.

Membrane potential fluctuations recorded from the soma of mature astrocytes are quite modest (i.e. within several millivolts) at best. The resting membrane potential of a typical astrocyte is less than −80 mV, which is close to the reversal potential of potassium (K^+^). Astrocytes have an order-of-magnitude lower input resistance than pyramidal cells owing to K^+^ channels that are permeable at resting membrane potentials (e.g. TWIK-1, TREK-1 and K_ir_4.1) [4,5] as well as the existence of hemichannels and gap junctions. While these properties and the lack of active conductance make astrocytes electrophysiologically quiescent, astrocytes have been reported to have cytosolic calcium (Ca^2+^) elevations and intercellular Ca^2+^ waves [2]. These Ca^2+^ elevations occur without large membrane potential changes, because the Ca^2+^ is released from internal Ca^2+^ stores such as the endoplasmic reticulum (ER). Such cytosolic Ca^2+^ elevations in astrocytes have also been described *in vivo* in rodents [6]. Triggers that initiate astrocytic Ca^2+^ elevation are diverse, but common neurotransmitters and neuromodulators are potent agonists for astrocytic Ca^2+^ elevation through G-protein coupled receptors (GPCRs). One of the key questions in neuron–astrocyte interactions is whether astrocytic Ca^2+^ elevations play any role in brain operation, and to identify the circumstances under which such neuron–glia interactions occur. In this article, we focus on how subcortical neuromodulatory signals mediate astrocyte–neuron interactions in the context of synaptic plasticity in cerebral cortical structures.

## 2. Volume transmission versus synaptic transmission

Chemical transmitters are released in two distinct transmission modes: wiring transmission and volume transmission (for classic reviews, see [7,8]). Wiring transmission is intercellular communication mediated via a physically defined connecting structure. Synaptic transmission is the primary mechanism of wiring transmission, and its primary feature is fast (millisecond-order) point-to-point communication. Glutamate and GABA are the predominant neurotransmitters for this in mammalian cortical structures. The potency and reliability of the synapse are the key determinants of information transmission. Astrocytic microprocesses that ensheath synapses are thought to increase the fidelity of synaptic transmission by rapid neurotransmitter clearance and insulation from other synapses [9].

**Figure 1.**
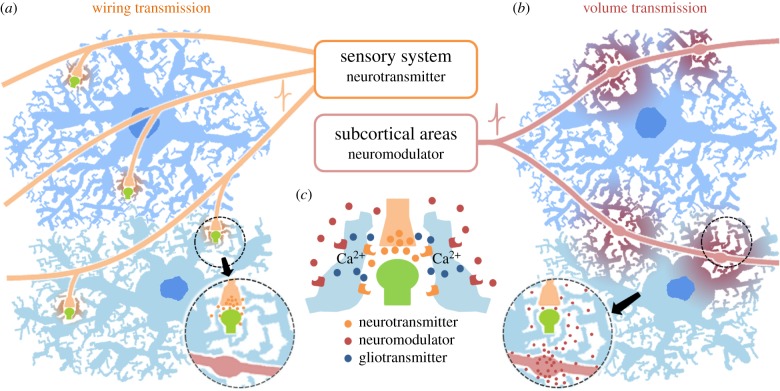
Wiring transmission versus volume transmission and their effects on astrocytes. (*a*) Wiring transmission targets designated synapses and produces localized responses in perisynaptic astrocytic processes. (*b*) In volume transmission, the neuromodulators diffuse into tortuous and convoluted ECS upon release from *en passant* varicosities. Such ECS diffusion results in activation of astrocytic GPCRs in larger areas than a synaptic component, resulting in synchronized and spatially spread astrocytic Ca^2+^ activities. (*c*) Volume transmission and synaptic transmission can occur simultaneously in brain states characterized by neuromodulator release, for instance, during attention.

Volume transmission is by non-synaptic release of neuromodulators diffusing through the extracellular space (ECS; figure 1), which is defined by an intricate and dense organization of synaptic and glial process morphology (for a review, see [10]). As a result, the manner of diffusion deviates significantly from free diffusion because of the tortuosity and limited volume fraction of the ECS. Subsequently, a relatively large number of cells sense neuromodulators via extrasynaptic receptors. In the cerebral cortex and hippocampus, volume-transmitted neuromodulators include acetylcholine and monoamines. The afferent fibres for neuromodulators are mainly of subcortical origin, and usually make asynaptic junctions in the cortex and hippocampus via terminal varicosities in stark contrast to glutamatergic and GABAergic innervation. For example, synaptic incidences are a mere 10–20% of the total varicosities for acetylcholine [11,12] and noradrenaline [13,14] and 20–30% for serotonin [15]. In addition to the complex ECS geometry, the true nature of ECS diffusion is complicated by the presence of diffusion obstacles (e.g. extracellular matrix and cell adhesion molecules) and active interference system (e.g. uptake by transporter or enzymatic degradation) [10,16]. Theoretical models and simulations have been compared with experimental data obtained by real-time iontophoresis or fluorescent macromolecule imaging [10].

As much as neurons receive this extrasynaptic neuromodulator transmission, astrocytes surrounding synapses are also the receivers. Serial reconstruction of the neuropil of rat hippocampal grey matter shows that glial processes occupy over 10% of all plasma membrane area [17]. This proportion is even higher when the analysis is confined to the extrasynaptic space, and thus glial surface represents a considerable target area for volume-transmitted neuromodulators. Moreover, astrocytes express receptors for subcortical neuromodulators [18].

Other remarkable differences between synaptic transmission and volume transmission are the time course and spatial range of signal transfer. While neurotransmitters travel 20–30 nm across the synaptic cleft, volume-transmitted neuromodulators travel on the scale of micrometres to reach their receptors. At the receptor end, ionotropic receptors dominate in glutamatergic and GABAergic synapses of the cerebral cortex and hippocampus, ensuring millisecond-order signal transmission. Extrasynaptic receptors of neurons include both ionotropic receptors and metabotropic GPCRs. Literature suggests that neuromodulator receptors in astrocytes are predominantly GPCRs that have a much slower signal transduction (at least hundreds of milliseconds) [19]. Therefore, while affecting many targets, volume transmission is not expected to provide temporally precise signal transmission. Considering the tight coupling of neuromodulatory systems and behavioural states, and the slow time course of GPCR signalling, elucidation of the significance of astrocytic activation by neuromodulators may yield a new insight into understanding neuronal information processing in distinct behavioural states.

## 3. Astrocytic response to neurotransmitters and neuromodulators

Astrocytes respond to neurotransmitters and neuromodulators through a wide variety of GPCRs. Their activations trigger production of inositol 1,4,5-triphosphate (IP_3_), which induces Ca^2+^ release from the ER. So far, several groups have reported that Ca^2+^ elevations in astrocytes lead to gliotransmission of glutamate, d-serine or ATP and in turn regulate neuronal activity and synaptic strength in brain slices [20–26]. d-Serine is an endogenous co-agonist of NMDA receptors (NMDARs), and several studies have suggested that astrocytes release d-serine by exocytosis [27–29]. d-Serine release from a single astrocyte can modulate neighbouring neuronal NMDAR currents [26], and basal astrocytic Ca^2+^ concentration and extracellular d-serine concentration are correlated [30]. Electron-microscopic analysis showed that glutamate and d-serine are localized in microvesicles near the ER within the perisynaptic processes of astrocytes [31], hinting at the significance of perisynaptic Ca^2+^ signalling in gliotransmission. Recent studies using high-resolution Ca^2+^ imaging of hippocampal slices suggested that astrocytic microprocesses respond to single synaptic activity with rapid and localized Ca^2+^ elevation [32,33]. Roles of gliotransmission from astrocytic processes in synaptic function have been studied in the hypothalamic nuclei, where the astrocytic coverage of synapses decreases during lactation. The availability of d-serine in the synapses was reduced in slices from lactating rats [34].

There is growing evidence that astrocyte-derived ATP, which was initially categorized as a paracrine messenger responsible for interglial propagation of Ca^2+^ waves [35–37], can regulate synaptic transmission [22,38]. Although several non-vesicular pathways have been identified, recent studies using transgenic mice selectively expressing a dominant-negative SNARE protein in astrocytes demonstrated the significance of vesicular release of astrocytic ATP [22,39]. Another study showed that electrical stimulation of excitatory input to the hypothalamus induces metabotropic glutamate receptor (mGluR)-dependent astrocytic Ca^2+^ elevation and release of ATP [40]. Notably, a rise in Ca^2+^ in the astrocyte compartments immediately adjacent to the postsynaptic neuron was necessary for ATP-mediated changes in synaptic transmission. Interestingly, noradrenaline application also led to astrocytic ATP release and similar synaptic transmission changes [41], implying that the astrocytes are capable of responding to and possibly integrating both neurotransmitters and neuromodulators (figure 1).

It has been shown that astrocytic Ca^2+^ signals in the adult brain are mediated by volume-transmitted neuromodulators. Upon electrical stimulation of locus coeruleus (LC; the sole source of noradrenergic input to cortex), astrocytes exhibit broad Ca^2+^ increases in somatosensory cortex [42]. Aversive stimulation, known to result in phasic LC activity, also led to widespread adrenergic astrocytic Ca^2+^ elevation throughout sensory cortex, which is more pronounced in awake conditions [43]. Acetylcholine also activates global astrocytic Ca^2+^ signalling *in vivo*. The predominant sources of cholinergic afferents for the cerebral cortex and hippocampus are the nucleus basalis of Meynert (NBM) and medial septum. We and others have demonstrated that stimulation of the respective cholinergic nuclei leads to muscarinic acetylcholine receptor (mAChR)-dependent astrocytic Ca^2+^ elevation in the cortex [44,45] and hippocampus [46]. Notably, NBM stimulation led to an increase in extracellular d-serine in the cortex of control mice, but not of mice lacking astrocyte-dominant IP_3_ receptors (IP_3_R2) [44]. Our results indicate that astrocytic Ca^2+^ responses by whisker or NBM stimulation differ in the following two aspects: (i) whisker stimulation induces mGluR-dependent weaker Ca^2+^ responses [47], whereas NBM stimulation produces mAChR-dependent robust responses; and (ii) while whisker-induced Ca^2+^ surges return to baseline even during stimulation, plateau Ca^2+^ increases persist throughout NBM stimulation.

GPCR signalling in astrocytes has also been suggested to regulate extracellular K^+^ [48,49], neurotransmitter uptake [50] and neurovascular coupling [51–53] (but also see [54,55]). On the other hand, some recent studies that use molecular genetics have challenged the validity of gliotransmission. Astrocytic expression of and subsequent activation of a foreign GPCR (MrgA1) to selectively induce astrocytic Ca^2+^ elevation [56,57] or genetic deletion of IP_3_R2s to diminish astrocytic Ca^2+^ elevations [57,58] did not result in a notable change in excitatory synaptic transmission in mouse hippocampal slices. This apparent contradiction may be due to the method used to stimulate astrocytes. For example, uncaging IP_3_ in MrgA1-positive astrocytes increased the frequency of glutamatergic miniature excitatory postsynaptic currents (mEPSCs) in nearby neurons [56]. A recent study further addressed this issue and showed that Ca^2+^ uncaging in astrocytes triggers glutamate release, whereas agonist activation of MrgA1, PAR-1 or purinergic receptors does not [49]. Moreover, astrocytic glutamate release can be mediated by channels [59,60], and Gq-coupled GPCRs may also have IP_3_-independent pathways [61]. Future investigation on neuromodulator-mediated Ca^2+^ signalling in astrocytic processes and their functional manipulation *in vivo* will advance our understanding of the role of astrocytes in normal brain function. Other key issues for future studies are to understand the functional significance of neuromodulator-driven global responses and neurotransmitter-driven individual localized transients and to identify the biological situation where these signalling modes are employed differentially or in synergy.

## 4. Neuromodulator activation and gamma oscillations

Distinct neuromodulators contribute to different modes of animals’ behavioural states. Likewise, animals’ behavioural states and neuronal population dynamics are tightly correlated. For instance, large amplitude slow waves (0.5–2 Hz) appear in the electroencephalogram (EEG) during deep sleep, whereas faster and lower amplitude patterns are seen in waking states. Gamma oscillations (30–100 Hz) appear during states of attention [62], and this rhythm is thought to bind neural representation of different sensory modalities [63]. Detailed cellular mechanisms underlying gamma oscillations are yet to be fully elucidated, but reciprocal interactions between excitatory pyramidal neurons and inhibitory interneurons, particularly parvalbumin positive fast-spiking basket cells, probably play a key role [64]. As attention is naturally related to cognitive processing and learning efficiency, synaptic plasticity is probably induced during the gamma oscillation state. Indeed, repetitive phase locked activity of neurons at a gamma frequency provides a situation favourable for spike-timing-dependent plasticity [65]. Moreover, this type of plasticity is enhanced by activation of mAChRs [65].

As neuromodulator release and EEG states are both highly correlated to an animal's behaviour, they should naturally be closely linked. As a matter of fact, the gamma states also coincide with release of neuromodulators. For instance, gamma oscillations are induced by electrical stimulation of the NBM in anaesthetized rats [66] or optogenetic stimulation of cholinergic neurons in the basal forebrain in awake mice [67]. Moreover, noradrenergic transmission has been shown to be crucial for waking gamma that appears shortly after gas anaesthesia wears off [68]. During awake and REM sleep periods, higher amounts of acetylcholine are released in the cortex and hippocampus than during slow wave sleep [69]. In accordance with cortical activation, cholinergic neurons in the basal forebrain increase their firing rates, and alter their firing mode from single spike to rhythmic bursting [70].

Exposure to enriched environments (EE) has been known to boost animals’ learning ability and its neural circuit remodelling effect has been studied for decades. We recently found that hippocampal gamma amplitude increases in rats raised in EE, which hints at a possible link between gamma oscillation and learning [71]. Increases of spine density and dendritic complexity are common effects of EE in the cortex and hippocampus. Although the mechanism for chronic gamma increase is most probably multifactorial, increased input to pyramidal cells is a conceivable factor, as gamma is a product of balanced excitatory and inhibitory synaptic input [72]. The requirement of NMDAR activation for chronic gamma enhancement [71] also suggests that a long-term potentiation (LTP)-like mechanism may be involved. Interestingly, GABAergic networks have also been reported to be altered by EE [73].

As well as neurotransmission, there are notable changes in neuromodulation after EE. Rats raised in EE after weaning show increased hippocampal and anterior cortical choline acetyltransferase activity after maze training [74]. Similarly, the concentration of released acetylcholine is higher when rats solve more difficult tasks [75]. The causal relationship between chronic gamma increase and neuromodulator systems is not resolved at this time. However, it is remarkable that many studies report enhanced LTP by neuromodulators including acetylcholine and noradrenaline, suggesting a permissive role of GPCR for synaptic plasticity and learning [76]. As described in §3, GPCRs are not only expressed in neurons. Given the existence and functional response of GPCRs in astrocytes, it is logical to ask whether activation of astrocytic GPCR has a role in synaptic plasticity *in vivo*.

## 5. Astrocytic modulation of synaptic plasticity during gamma states

As gamma states coincide with volume transmission of neuromodulators including acetylcholine and noradrenaline, astrocytic Ca^2+^ dynamics are more active during these states (§3). Recently, three independent studies have investigated the role of gamma-state-induced astrocytic Ca^2+^ elevation in synaptic plasticity. These studies were performed in the somatosensory cortex [44], visual cortex [45] and hippocampus [46]. In each of these studies, the respective cholinergic nucleus was stimulated while sensory stimuli or electrical afferent stimulation was presented to anaesthetized animals. As a result, long-lasting enhancements (more than 1 h) in stimulus-evoked potential or neuronal firing rate were observed. These effects were diminished in IP_3_R2 knockout (IP_3_R2-KO) mice, in which astrocytic large Ca^2+^ elevations are deficient, suggesting the causal relationship between the astrocytic Ca^2+^ elevation and induction of the synaptic plasticity.

NBM-evoked cortical gamma oscillations seem to be uninfluenced by astrocytic Ca^2+^, as the duration of gamma oscillations was similar between wild-type and IP_3_R2-KO mice [44]. Neuronal activity is temporally coordinated in gamma rhythms by NBM stimulation and this synchronization could be a prevailing mechanism of augmented synaptic plasticity [77]. However, the deficiency of NBM-associated cortical plasticity in IP_3_R2-KO mice strongly supports a role of astrocytic Ca^2+^ signalling in the synaptic plasticity.

In our investigation on plasticity in the somatosensory cortex, astrocytic Ca^2+^ activities were elevated during costimulation of whiskers and NBM. Similarly, the extracellular concentration of d-serine is elevated during NBM stimulation and returns to the baseline thereafter. Considering the major role of NMDARs in LTP [78], the lack of extracellular d-serine increase in IP_3_R2-KO mice suggests a pivotal role of astrocytic Ca^2+^ signalling during the induction phase of the synaptic plasticity. Chen *et al.* [45] showed that single unit activities in the visual cortex are enhanced when visual orientation stimuli are combined with NBM stimulation. Importantly, the neuronal response is enhanced only for the orientation paired with NBM stimulation. As the orientation tunings of individual synapses are intermingled in mouse primary visual cortex [79], this result advocates the importance of sensory input as the determinant for specificity of plasticity. Further investigation on spatio-temporal relationship of active astrocytes and augmented synapses should characterize the effective range of gliotransmission.

**Figure 2.**
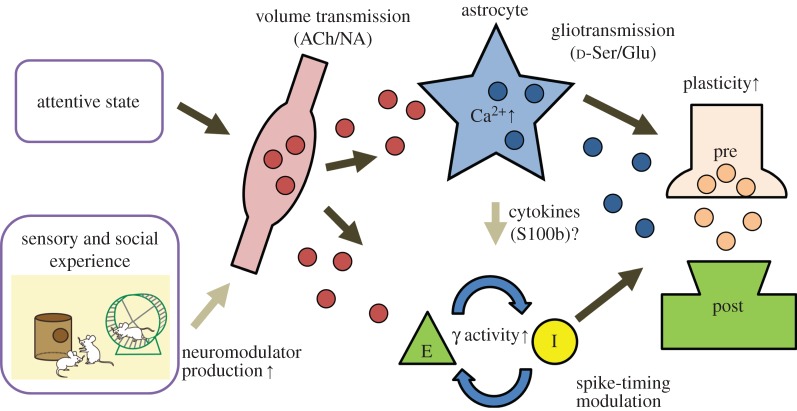
Schematic diagram for neuron–astrocyte interaction in the context of gamma-state-induced synaptic plasticity. Attentive states drive volume transmission of subcortical neuromodulators which in turn activates neuronal gamma oscillations and astrocytic gliotransmission to establish a state for synaptic plasticity induction. Sensory and social experience enhances neuromodulator production and gliotransmission of cytokines enhances gamma oscillations, although the exact mechanism remains to be elucidated. ACh, acetylcholine; NA, noradrenaline; E, excitatory neuron; I, inhibitory neuron.

While the synaptic plasticity in our study is NMDAR-dependent, a study by Navarrete *et al.* [46] investigated cholinergically augmented hippocampal CA3–CA1 plasticity in the presence of an NMDAR blocker. Their results suggest that glutamate acts as the gliotransmitter affecting neuronal mGluRs to express presynaptic plasticity, whereas a recent paper suggests other interpretations such as transient change of extracellular ionic composition [48]. These differences suggest that the molecular mechanisms of astrocyte-assisted synaptic plasticity may be diverse, but the common denominator of all *in vivo* experiments is the activation of cholinergic volume transmission (figure 2). Notably, similar hippocampal plasticity was evoked when medial septum stimulation was replaced by tail pinch [46], and atropine could largely, but not completely, block the field response potentiation, suggesting that other neuromodulators such as noradrenaline could also be involved. Additionally, in prolonged gamma states, astrocytes can secrete cytokines including S100B and influence network synchronization [80] and synaptic plasticity [81].

## 6. Concluding remarks

We have discussed a possible role of cortical astrocytes as an element enhancing cortical plasticity via gliotransmission. Volume transmission of subcortical neuromodulators serves as the drive for activation of astrocytes and gamma oscillations. Gamma oscillations appear during attentive states and provide temporally synchronized activation of groups of neurons (i.e. cell assembly), association of which will lead to formation of memory and learning. Considering the astrocytic expression of functional GPCRs for neuromodulators and the tight relationship between the subcortical neuromodulator system and the cognitive states of an animal, the framework of this model is sound. Recently, multiple groups have shown that the cholinergic system can mediate such a mechanism in rodent cortex and hippocampus [44–46,82]. Remarkably, noradrenergic transmission is reported to provide a dominant drive for astrocytic Ca^2+^ elevations during awake states [43]. Cholinergic volume transmission may provide an additional input to enhance Ca^2+^ elevations in astrocytes during attention. Indeed, a synergistic effect of acetylcholine and noradrenaline in synaptic plasticity has been described [83,84]. It is conceivable that similar operating principles are in effect in extracortical areas. For instance, the basal ganglia system is under the strong control of dopaminergic innervation, whereas the cerebellar cortex receives significant serotonergic and noradrenergic innervations. Molecular and physiological investigations on the heterogeneity of astrocytes will be important to understand the regional operational characteristics of astrocyte–neuron interactions. Response to neuromodulators is widespread across the astrocytic syncytium owing to the nature of volume transmission, and possibly owing to interastrocytic Ca^2+^ wave propagation [85]. Synaptic activity-driven elevation of focal Ca^2+^ rise in astrocytes [32,33] may provide us an additional mechanism to promote synaptic efficacy.
